# Hepatitis B Virus and B-cell lymphoma: evidence, unmet need, clinical impact, and opportunities

**DOI:** 10.3389/fonc.2023.1275800

**Published:** 2023-10-20

**Authors:** Maya Rosenberg, Maria Poluch, Colin Thomas, Paola Sindaco, Alan Khoo, Pierluigi Porcu

**Affiliations:** ^1^ Department of Internal Medicine, New York University Langone Health, New York, NY, United States; ^2^ Sidney Kimmel Medical College, Thomas Jefferson University, Philadelphia, PA, United States; ^3^ Department of Medical Oncology, Hospital of the University of Pennsylvania, Philadelphia, PA, United States; ^4^ Sidney Kimmel Cancer Center, Thomas Jefferson University, Philadelphia, PA, United States; ^5^ Department of Medical Oncology, Thomas Jefferson University, Philadelphia, PA, United States

**Keywords:** hepatitis B, B-cell, lymphoma, DLBCL, double hit, viral oncogenesis

## Abstract

Nearly a billion people worldwide are infected with the hepatitis B Virus (HBV) and about a third of them have chronic infection. HBV is an important cause of morbidity and mortality, including acute and chronic hepatitis and hepatocellular carcinoma (HCC). Screening and control of primary HBV infection through vaccination represent a major advance in global public health, but large sections of the world population, in both developed and underdeveloped countries, remain unscreened and unvaccinated. In addition to being a global cause of liver disease, an important role of HBV in lymphoma has also emerged. First, the high risk of HBV reactivation in previously infected patients receiving chemo-immunotherapy necessitates the systematic evaluation of HBV serological status in all non-Hodgkin’s lymphoma (NHL) cases and preemptive antiviral therapy for those who may have chronic or occult HBV infection. Second, HBV has been shown to infect lymphocytes, namely B-cells, and has been associated with a higher risk of developing B-cell lymphoma, most clearly in countries where HBV is endemic. While the risk of HBV reactivation with chemoimmunotherapy in NHL is well known, the role and the impact of HBV as a global lymphoma risk factor and potential oncogenic driver in B-cells are very poorly understood. Here, we review the clinical and scientific evidence supporting an association between HBV and B-cell lymphoma, with a particular focus on diffuse large B-cell lymphoma (DLBCL) and provide an overview of the estimated impact of HBV infection on the biology and clinical course of DLBCL. We also discuss ways to gain a better insight into the unmet need posed by HBV in lymphoma and whether assessing immune responses to HBV, measuring viral loads, and detecting the presence of HBV-encoded proteins in tumor tissue could be integrated into the molecular and clinical risk stratification of patients with DLBCL.

## 1 Introduction

Viral infections are associated with several types of cancer with widely different levels of risk in different populations and geographical areas and the global role of viruses as oncogenic drivers is widely recognized. Six viruses (HBV, Hepatitis C Virus [HCV], Epstein-Barr Virus [EBV], Human papillomavirus [HPV], Human T-Cell Lymphotropic Virus [HTLV-1], and Human Herpes Virus-8 [HHV-8]) are classified as class I carcinogenic agents by the International Agency for Research on Cancer (IARC) (1) and about 10% of all cancers worldwide can be attributed to viruses (2). Global vaccination campaigns for HPV and HBV have been implemented, and efforts to test the efficacy and safety of vaccines for EBV are ongoing in clinical trials (NCT04645147, NCT05164094) (3). In addition, ongoing studies show the potential of targeted therapies for virus-associated cancers. EBV-targeting adoptive cellular immunotherapies (4) and new treatments that use the presence of oncogenic viruses as an intrinsic tumor-specific vulnerability are being investigated. For example, a small molecule inhibiting the EBV protein EBNA-1 (VK-2019) has shown promising anti-tumor efficacy in preclinical models of EBV-positive cancers (5) and a Phase I clinical trial of VK-2019 in nasopharyngeal carcinoma (NPC) is ongoing (NCT04925544). Additionally, the combination of the histone deacetylase (HDAC) inhibitor Nanatinostat and the nucleoside analog valganciclovir (VGCV) was recently granted orphan drug designation (ODD) for EBV-positive lymphomas by the FDA, based on initial Phase 1/2 data (6), and an international multi-cohort Phase 2 clinical trial (Naval-1) is ongoing (NCT05011058) (7). These studies show the potential of developing targeted therapies for virus-associated cancers.

Awareness of the linkage between carcinogenic viruses and cancers remains inadequate in the public and in parts of the medical and advocacy communities. This is the case of the association between prior infection with HBV and risk of B-cell lymphoma, in particular diffuse large B-cell lymphoma (DLBCL), the most common type of aggressive B-cell lymphoma worldwide (1). While the importance of chronic HBV infection in the development of HCC is well established, its role as a risk factor for DLBCL is less known. Consequently, education efforts to increase awareness of symptoms of DLBCL among HBV seropositive patients are inadequate, potentially leading to a delay in diagnosis of DLBCL in this patient population. Likewise, the impact of HBV vaccination and antiviral therapy on the risk of developing DLBCL remains unknown.

The goal of this paper is to provide a background on HBV infection and DLBCL, and then critically review the evidence supporting a role for chronic HBV infection in DLBCL, outline the criteria currently defining HBV-associated DLBCL, provide available estimates of its frequency and distribution globally and in the U.S., and review the distinctive aspects of HBV-associated DLBCL that have been identified. We will also offer an assessment of the unmet need and opportunity in terms of scientific discovery and public health impact.

## 2 Background

### 2.1 Hepatitis B Virus

#### 2.1.1 HBV as a global health problem

In the United States, 850,000 individuals are estimated to be living with HBV (8). The prevalence of past or present HBV infection amongst people in the U.S. is 4.3% (9). However, populations of foreign-born minorities, with a higher prevalence of HBV, are likely underrepresented in this calculation. This pathogen is thought to be responsible for over 296 million chronic infections worldwide (10). Further, in 2021, the World Health Organization (WHO) reported that only 30.4 million people living with HBV knew their HBV status, accounting for only 10% of the total people in the world living with chronic HBV infection (10).

HBV infection carries a heavy financial toll for patients and healthcare systems, with total HBV hospitalization charges in the U.S. increasing from $357 million in 1990 to $1.5 billion in 2003 (11). In 2019, the total mean all-cause annual healthcare costs for HBV patients with Medicare who had decompensated cirrhosis, HCC, received liver transplants, or had compensated liver disease was $479,595 (12). This financial burden is felt globally (13). The significant financial and health impact of HBV makes understanding this virus and its sequelae important.

#### 2.1.2 HBV’s life cycle

HBV is an enveloped virus with a circular, partially double-stranded DNA genome, which belongs to the *Hepadnaviridae* family. The infectious virion consists of a lipid envelope containing the HBV surface antigen (HBsAg). This surrounds an inner nucleocapsid composed of the HBV core antigen (HBcAg) complexed with virally encoded polymerase (14). The viral genome contains 4 overlapping open reading frames that encode proteins essential for viral replication (14). HBV entry into host cells is mediated by low-affinity binding to heparan sulfate proteoglycans (HSPGs), followed by high-affinity binding to sodium taurocholate co-transporting polypeptides (NTCPs). Glypican 5 is an HSPG that preferentially binds HBV (15, 16). HSPGs are found at the surface and in the extracellular matrix of most human cells. At least some of the virus’ hepatotropic nature is thought to be due to the prevalence of glypican 5 in the liver.

#### 2.1.3 Infection, clinical manifestations, and outcomes

Worldwide, the most common route of HBV transmission is perinatal transmission, especially in endemic areas (15). HBV is also transmitted through percutaneous and mucous membrane exposures and sexual intercourse with infected individuals (8). Once the infection is acquired, the host can experience an acute infection with complete recovery, a fulminant course with hepatic failure, or a chronic infection (17).

The infection is diagnosed by detecting HBsAg and anti-hepatitis B core IgM antibody (HBcAb) in plasma. Chronic HBV is characterized by the persistent presence of HBsAg in the serum for greater than 6 months, in addition to HBcAb (18). Chronic HBV infections are typically characterized by four phases, varying in length and defined by laboratory results and clinical symptoms.

Mortality among adults with chronic HBV far exceeds that of uninfected individuals. One study including 39,206 patients concluded that those with chronic HBV infection had a 1.9-fold (95% CI 1.1–3.3) increased hazard of all-cause mortality compared to uninfected people, and a 13.3-fold (95% CI 3.9–45.5) increased hazard of liver-related mortality (19). Hepatocellular carcinoma (HCC) is closely associated with HBV, with studies showing that chronically infected individuals have 100 times the risk of developing HCC than non-carriers (20). Unfortunately, deaths from chronic HBV infection are increasing, with a rising incidence of HCC of particular concern in endemic areas like Africa and the Western Pacific (21). Professional medical societies, such as World Health Organization (WHO) and the American Association for the Study of Liver Diseases (AASLD), vary in their recommendations for screening and treatment (22). There are significant barriers to effective HBV screening, prevention, and treatment, with an impact that may not be limited to chronic disease and HCC, but may include B-cell lymphoma, specifically diffuse large B-cell lymphoma (DLBCL).

### 2.2 Diffuse large B-cell lymphoma

Non-Hodgkin’s Lymphomas (NHLs) are hematologic malignancies of mature lymphocytes and one of the more common cancers in the United States, accounting for about 4% of all cancers (23). DLBCL is an aggressive NHL that comprises about 30% of all lymphoma cases. It is the most common subtype of NHL in the U.S (24) and worldwide (25). DLBCL is most prevalent in elderly patients, with a median age at diagnosis in the 7^th^ decade of life. The incidence of DLBCL varies by race, with racial differences in age and gender distribution (26–28). However, the incidence of DLBCL increases with age for all races (27).

In clinical practice, DLBCL is frequently classified based on immunohistochemistry (IHC)-defined cell-of-origin (COO). There are two major subtypes, a germinal center B-cell (GCB) type and a non-GCB type, corresponding to the activated B-cell (ABC) type defined by gene expression profiling (29). The use of cytogenetics and fluorescence *in situ* hybridization (FISH) further classify DLBCL by identifying chromosomal translocations in tumor cells, in particular rearrangements involving *MYC* (8q24), BCL-2 (18q21), and BCL-6 (3q27). DLBCL carrying genetic rearrangements of both *MYC* and *BCL-2* genes, with or without a rearranged BCL-6 gene, were formerly known as double-hit (or triple-hit if BCL-6 is also involved) lymphomas. Double hit and triple hit (DH/TH) DLBCL represent approximately 10% of all DLBCL and are particularly aggressive and chemotherapy-resistant. Data are emerging about the impact of deletions or inactivating mutations of *TP53*, a tumor suppressor gene involved in cell cycle arrest and apoptosis. *TP53* mutations are present in about 10% of DLBCL cases and are an independent predictor of poor prognosis (30, 31). More recently, next generation sequencing (NGS) studies of whole genomes and transcriptomes in untreated DLBCL have identified several genetic clusters and molecular subgroups characterized by specific cancer-driving signatures and epigenetic pathways, with distinct outcomes (32–34).

The International Prognostic Index (IPI) is a risk stratification tool for DLBCL patients taking into account age, performance status, serum lactate dehydrogenase (LDH), number of extranodal sites, and stage (35). This scale, or one of its modifications, is the mainstay of risk stratification in DLBCL (36). While multiple studies report inferior outcomes in patients with EBV-positive (37), HCV-positive (38), and HBV-positive lymphomas (39), serological status and quantitative viral load measurements have not been included in any of the risk-stratification tools for lymphoma, except for plasma EBV DNA in extranodal NK/T-cell lymphoma (ENKTL) (40).

The standard first-line therapy for most DLBCL patients is the combination of the chemotherapy regimen CHOP (cyclophosphamide, doxorubicin, vincristine, and prednisone), with rituximab, a monoclonal antibody that targets the pan-B cell surface antigen CD20. This regimen is referred to as R-CHOP. With this regimen, 60-65% of patients are cured. The prognosis is poor for 35-40% of patients who relapse following R-CHOP or have refractory disease. Current research is focused on finding better risk-stratification tools, to identify patients who will do well with R-CHOP versus those who may require more aggressive regimens up-front.

Patients with DH/TH DLBCL generally have more aggressive clinical courses, with advanced-stage presentation, extranodal involvement, higher serum LDH, and a high IPI score. DH/TH DLBCL carries a particularly poor prognosis, with a 5-year survival of <30% (41). *TP53* is also commonly mutated in DH/TH DLBCL and Double-Hit Signature (DHITsig)-positive DLBCL, which adds an unfavorable feature to these patients (42). Therapeutic approaches more aggressive than R-CHOP are often used for DH DLBCL, but overall survival rates remain poor (43).

## 3 Association between hepatitis B Virus and B-cell lymphomas

### 3.1 Hepatitis B Virus and non-Hodgkin’s lymphoma

**Figure 1:**
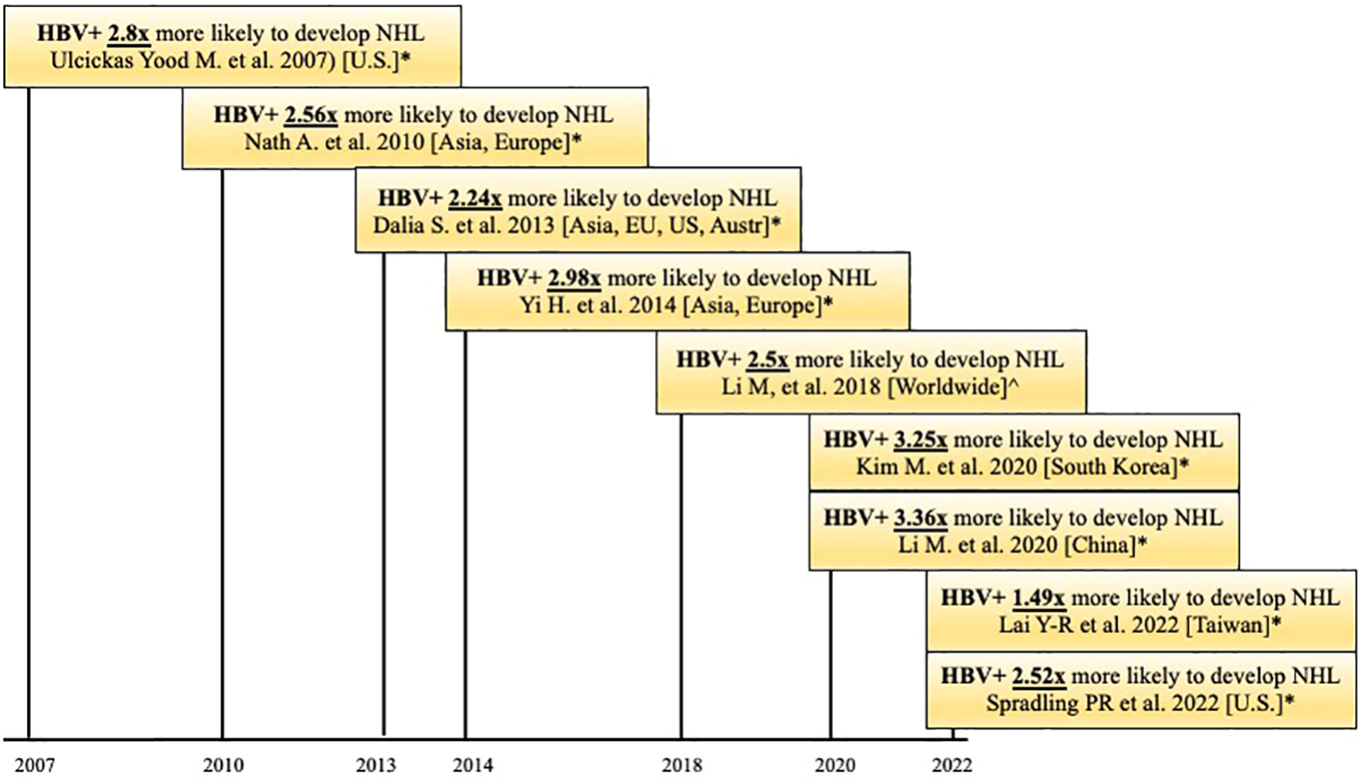
Timeline of published case-control and cohort studies (*) and meta-analyses (^) that observed that HBV patients are more likely to develop Non-Hodgkin Lymphoma (NHL). HBV+ = positive for HBsAg.

It is well known that HBV increases the risk of HCC. Studies show that chronically HBV-infected individuals have 100 times the risk of developing HCC than non-infected individuals (20). Epidemiological and seroprevalence studies have shown that HBV also increases the risk of other types of cancer, such as NHL. This association, particularly between HBV and B-cell lymphomas, has been documented in studies from both endemic and non-endemic areas. A 2007 case-control study conducted in the U.S. showed that patients (N=3,888) with chronic HBV infection were 2.8 times more likely to develop NHL than matched controls (N=205,203; HR = 2.80, 95% CI = 1.16-6.75). This study controlled for age, race, sex, income, Charlson comorbidity index, study site, and HCV infection (44). A recent 2018 meta-analysis of 58 published studies included data on 53,714 NHL cases and 1,778,591 controls. The studies were from Asia (N=46), Europe (N=8), North America (N=2), Africa (N=1) and Oceania (N=1). The meta-analysis found that HBV-infected individuals were 2.5 times more likely to develop NHL than non-infected individuals (45). These findings have been replicated (46, 47). A recent cohort study by Spradling (47) used data from the U.S. National Cancer Institute (NCI) and the National Program of Cancer Registries to assess the incidence of specific cancers in patients ≥20 years old with HBV compared to non-HBV-infected patients of the same age range. Patients with previous or current HCV or Human Immunodeficiency Virus (HIV) coinfection were excluded. Results showed, with a 95% confidence interval, that patients with HBV had over 2.5 times the risk of developing NHL compared to the general population. Thus, multiple case-control and cohort studies from endemic and non-endemic areas show that HBV infection is associated with a 2-3 fold higher risk of developing NHL, compared to controls, leading to the question of the mechanism behind this association (**Table 1**, **Figure 1**).

### 3.2 HBV’s Lymphotropism

The ability of HBV to infect human lymphocytes has been reported in multiple studies but remains poorly understood (**Table 1**). A 2011 study used human bone marrow (BM) from the iliac crest of healthy volunteers aged 18-36 years, who had no serologic evidence of current or previous HBV infection. BM cells were exposed to HBV *in vitro* for 24 hours. Following a ten-week incubation period, the BM stem cells were harvested, and their DNA was extracted. HBsAg and HBeAg levels were then measured using electrochemiluminescence (56). This approach assessed the efficiency with which HBV productively infected bone marrow stem cells *in vitro*, based on the expression of HBV-encoded proteins. Results showed that the infection efficiency was comparable to its ability to infect primary human hepatocytes and human hepatoma cell lines (56). Later studies confirmed this finding (57). It was further shown that the HBV residing in a patient’s BM stem cells could infect HBV-naïve hepatocytes in transplanted livers. This finding was important as it presented a possible source of graft re-infection following stem cell transplantation in patients with chronic HBV (58, 59).

**Table 1:** Summary of studies analyzing HBV and its effect on Non-Hodgkin Lymphoma (NHL) and Diffuse Large B-Cell Lymphoma (DLBCL).

Study (Year)	Location	Sample	Key Findings
An J, Kim JW, Shim JH, et al. (2018) (48)	South Korea	95,034 patients with non-hepatocellular malignancy, 118,891 controls	HBV was positively associated with DLBCL (AOR 1.75, p = 0.003 for men, AOR 4.37, p < 0.001 for women) when compared to other B-NHLs
Cheng C-L, Huang S-C, Chen J-H, et al. (2020) (49)	Taiwan	416 DLBCL cases	Compared with DLBCL patients who were HBsAg-negative, HBsAg-positive patients had a lower overall response rate (ORR) (76.5% vs. 85.5%, p = .043), poorer 5-year overall survival (OS) rate (57.2% vs. 73.5%, p <.001), and shorter 5-year progression-free survival (PFS) rate (47.2% vs. 60.7%, p = .013).
Dalia S, Chavez J, Castillo JJ, Sokol L. (2013) (50)	Asia, Australia, Europe, U.S.	1,377 NHL cases and 2,633,274 controls	HBV was positively associated with all NHL subsets when compared with the control population (OR 2.24; 95% CI 1.80 – 2.78; p ¾ 0.001). HBV was positively associated with DLBCL specifically (OR 2.05; 95% CI 1.25 – 3.35; p ¾ 0.001)
Kim M, Lee YK, Park B, Oh DJ, Choi HG. (2020) (51)	South Korea	929 NHL cases, 3716 controls	HBV rates were higher in the NHL group than in the control group (OR 3.25; 95% CI 1.99 – 5.31; p < 0.001)
Lai Y-R, Chang YL, Lee CH, Tsai TH, Huang KH, Lee CY. (2022) (52)	Taiwan	54,157 HBV or HCV cases and 270,785 controls	Incidence of NHL was significantly higher in patients with HBV than in patients from the general population (HR 1.49; 95% CI 1.94 – 3.19)
Li M, Gan Y, Fan C, et al. (2018) (45)	Africa, Asia, Europe, North America, Oceania	53,714 NHL cases and 1,778,591 controls.	HBV infected individuals were 2.5 times more likely to develop NHL than non-infected individuals. (95% CI 2.20 – 2.83)
Li M, Shen Y, Chen Y, et al. (2020) (53)	China	411 NHL cases, 957 controls	Positive rates of HBsAg (OR 3.11; 95% CI 2.20 – 4.41) and HBeAg (OR 3.99; 95% CI 1.73 0 9.91) were significantly higher in patients with NHL. Prevalence of HBsAg was significantly increased in B NHL (OR 3.36; 95% CI 2.33 – 4.84) but not in T-cell NHL.
Mahale P, Engels EA, Koshiol J. (2019) (46)	U.S.	1,825,316 first cancer diagnoses and 200,000 controls	HBV was positively associated with DLBCL (OR 1.24; 95% CI 1.06 – 1.46)
Nath A, Agarwal R, Malhotra P, Varma S. (2010) (54)	China, Egypt, Italy, Japan, Romania, Saudi Arabia, Singapore, South Korea, Turkey	3,262 NHL patients with 1,523,205 controls and 3,888 HBV patients with 205,203 controls	HBV was positively associated with NHL when compared with the control population (OR 2.56; 95% CI 2.24 – 2.92)
Spradling PR, Xing J, Zhong Y, et al. (2022) (47)	U.S.	5,773 HBV cases	Compared with the general population, substantially higher incidence among HBV-infected patients was observed for NHL (SRR 2.52)
Ulcickas Yood M, Quesenberry CP, Guo D, et al. (2007) (44)	U.S.	3,888 chronic HBV patients and 205,203 controls	Patients with chronic HBV infection were 2.8 times more likely to develop NHL than matched controls (HR = 2.80, 95% CI = 1.16-6.75)
Yi H, Chen JJ, Cen H, Yan W, Tan XH. (2014) (55)	China, Denmark, France, Germany, Greece, Italy, Netherlands, Norway, South Korea, Spain, Sweden, United Kingdom	5,396 B NHL cases and 20,671 controls	HBV was positively associated with B-NHL when compared with control population (OR 2.98; 95% CI 2.30 – 3.86)

During HBV’s life cycle, viral DNA can be integrated into the genome of infected cells (60). Integrated genomes can serve as templates for RNAs coding for viral proteins. HBV genome integration is a primary driver of HCC. Known cancer genes such as the telomerase reverse transcriptase (TERT) (61), mixed-lineage leukemia 4 (MLL4) (62), and Cyclin E1 (CCNE1) (63) are preferential integration sites in HCC and about one-third of the genes recurrently targeted by HBV integration are cancer-related genes (64). Recently, Svicher et al. published an overview of the mechanisms of HBV DNA integration into immune cells, highlighting the hypothesis that the oncogenic effect of HBV in lymphoma is driven by the integration of HBV DNA into lymphocytes (65). It was noted that peripheral blood mononuclear cells (PBMCs) may act as a extrahepatic reservoir for HBV infection.

**Figure 2:**
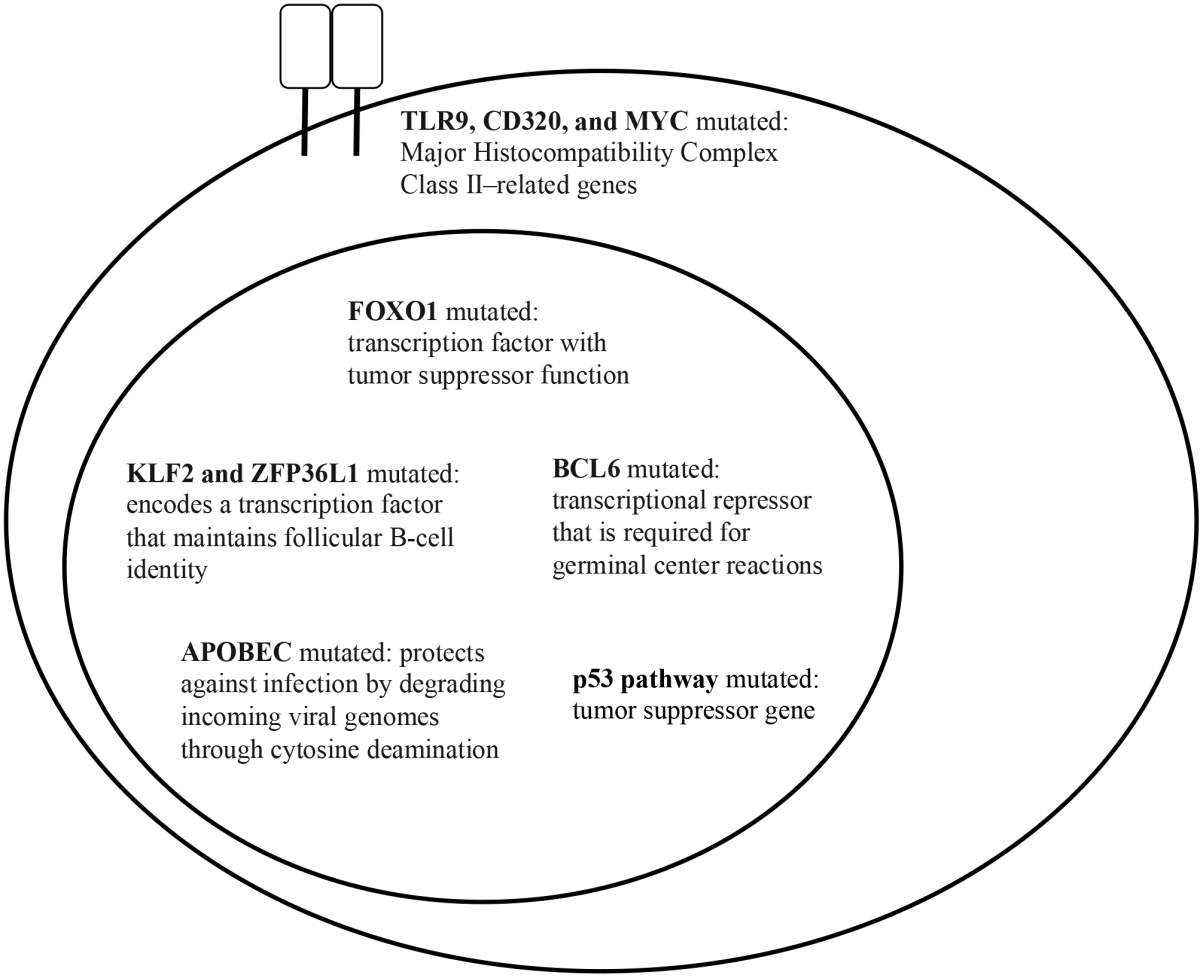
HBsAg+ DLBCL key gene mutations (data taken from Ren W. et al. (39)).

HBV DNA integration has been shown to affect multiple gene sites (65), as shown in **Figure 2**. A 2020 study identified the integration of HBV DNA in the lymphoma cells of 34 individuals with NHL (53). In total, 313 integration sites were identified. Half of the integration occurred in intergenic regions (49.5%), and the remaining took place in introns (44.7%), 3’-untranslated region (1.6%), gene upstream region (1.3%), and gene downstream region (1.3%). In the NHL samples, HBV integration had preferential targets. Seven genes, *ANKS1B*, *CAPZB*, *CTNNA3*, *EGFLAM*, *FHOD3*, *HDAC4*, and *OPCML* were found to be repeatedly targeted by HBV DNA integration. HBV DNA is regarded as a strong cis activator of flanking genes, so integration can influence the expression of target genes over a long distance (60). Six of the seven genes were found to be overexpressed in NHL, based on publicly available databases such as TCGA. KEGG (Kyoto Encyclopedia of Genes and Genomes) analysis of the HBV-targeted genes in NHL revealed that terms related to developmental process and cell differentiation, signal transduction, cell junction, and transcriptional regulation were significantly enriched (p<.05). Axon guidance was the most impacted pathway (p<0.0001), followed by Ras signaling, glycosaminoglycan biosynthesis, and cytokine-cytokine receptor interaction (p<.05). These pathways are also enriched in studies of HBV-targeted genes in HCC which implies that some pathways are commonly affected by HBV integration in HCC and NHL. This study is important, as it demonstrates that HBV DNA can integrate into the genome of NHL cells, affecting genes that have been reported to play an oncogenic role in other cancers.

Numerous *in vitro* and *in vivo* studies have shown the lymphotropism of HBV. *In vitro*, HBV was shown to be able to infect bone marrow progenitor cells and inhibit their growth (66). Mature lymphocytes were also shown to be infected with HBV, with HBV mRNA found in B-cells and T-cells (67). *In vivo* studies, most notably with chimpanzees, showed that chimpanzees with chronic HBV infection showed HBV DNA in PMBCs (68). Svicher et. al. further discusses the evidence that HBV infection is persistent in hematopoietic and lymphoid cells, which may act as a site for HBV reactivation and genome changes (65).

### 3.3 HBV and DLBCL

Multiple retrospective studies and meta-analyses have shown that in patients chronically infected with HBV, the most common type of NHL is DLBCL (16, 39, 50, 54, 55). HBV-associated DLBCL has been shown to have an incidence of 14.3% in West Africa and, according to a meta-analysis, has poor prognosis (69, 70). A 2018 study by An et al. (48) revealed a significantly positive link between HBV infection and DLBCL (adjusted odds ratio [AOR] 1.75, p = 0.003 for men, and AOR 4.37, p < 0.001 for women) when compared to other B-NHL. A 2020 study by Cheng et al. (49), including 426 patients with DLBCL at the National Taiwan University Hospital in Taipei, Taiwan, found that 23.6% of the patients were positive for HBsAg. When compared to HBsAg- patients, HBsAg+ patients were younger, diagnosed more frequently with advanced-stage disease, had lower overall response rates to R-CHOP (57.2% vs 73.5%, p < 0.001), and had shorter 5-year progression-free survival rates (47.2% vs 60.7%, p=0.013) (49). Another 2020 study, including data from 929 patients with NHL and 3716 healthy subjects in Korea, found that HBV rates were higher in the NHL group than in the control group (p < 0.001). The adjusted OR of HBV infection in patients with NHL was 3.25 (95% CI, 1.99 – 5.31) (51). Lastly, a 2022 retrospective cohort study used the nationally representative database in Taiwan to investigate the correlation between HBV and NHL. The study showed that the incidence rate of NHL was significantly higher in patients with HBV than in patients from the general population (HR, 2.49; 95% CI, 1.94 – 3.19) (52) (**Table 2**).

**Table 2:** Summary of studies showing differential outcomes in HBV-positive and HBV-negative DLBCL patients.

Study (Year)	Location	Sample	Key Findings
Cheng C-L, Huang S-C, Chen J-H, et al. (2020) (49)	Taiwan	416 DLBCL cases	HBsAg+ patients were younger and diagnosed more frequently with advanced stage disease; these patients had lower ORR to R-CHOP (57.2% vs 73.5%, p < 0.001) and shorter 5-year PFS rates (47.2% vs 60.7%, p=0.013), compared to HBsAg- patients
Li M, Shen Y, Chen Y, et al. (2020) (53)	China	411 NHL cases, 957 controls	HBV patients had a significantly higher level of serum LDH (p < 0.001), a more advanced stage of NHL (p = 0.001), a worse ECOG performance status (p = 0.029), and a less favorable prognosis (p = 0.023).
Ren W, Ye X, Su H, et al. (2018) (39)	China	275 DLBCL cases	DLBCL patients with concomitant HBV infection were characterized by a younger age (median age, 42 vs 60 years; p <.0001), a more advanced disease stage at diagnosis (p = 0.0002), higher international prognostic index (p = 0.007) and reduced overall survival.

HBV proteins can be detected in tissue biopsies from patients with DLBCL and chronic HBV infection. Huang et al. assessed tumor biopsies from 96 HBsAg+ and 10 HBsAg- DLBCL patients treated at five Chinese centers (16). The HBV antigen HBx, a protein essential for viral replication, was present in the lymphoma cells in 48.9% of HBsAg+ DLBCL patients; additionally, the HBV antigen Pre-S2, a component of HBsAg, was detected in the lymphoma cells of 57.2% HBsAg+ DLBCL patients. The authors also showed that the presence of HBx antigen in DLBCL cells was associated with high MYC expression (**Table 3**).

**Table 3:** Summary of studies showing integration of HBV genome/expression of HBV genes in lymphoma.

Study (Year)	Location	Sample	Key Finding
Lau KC, Joshi SS, Gao S, et al. (2020) (57)	China, U.S.	52 HBV cases	The replicative potential of HBV within lymphoid cells was evidenced by up-regulation of viral DNA in peripheral blood mononuclear cells (PBMC) supernatant after ex vivo mitogen-stimulation. Increased viral replication was evidenced by increased levels of HBV cccDNA and enhanced viral mRNA expression.
Li M, Shen Y, Chen Y, et al. (2020) (53)	China	411 NHL cases, 957 controls	HBsAg, HBcAg, and HBV DNA were detected in 34.4%, 45.2%, and 47.0% of the NHL tissues, respectively. There was a total of 313 HBV integration sites isolated from the NHL tissues. Terms related to developmental process and cell differentiation, signal transduction, cell junction, and transcriptional regulation were significantly enriched (p<.05). Axon guidance was the most impacted pathway (p<0.0001), followed by Ras signaling, glycosaminoglycan biosynthesis, and cytokine-cytokine receptor interaction (p<.05). These pathways are also enriched in studies of HBV-targeted genes in HCC which implies that some pathways are commonly affected by HBV integration in HCC and NHL.
Ma R, Xing Q, Shao L, et al. (2011) (56)	–––	–––	Results showed that the ability of HBV to infect bone marrow stem cells *in vitro* was comparable to its ability to infect primary human hepatocytes and human hepatoma cell lines.

**Figure 3:**
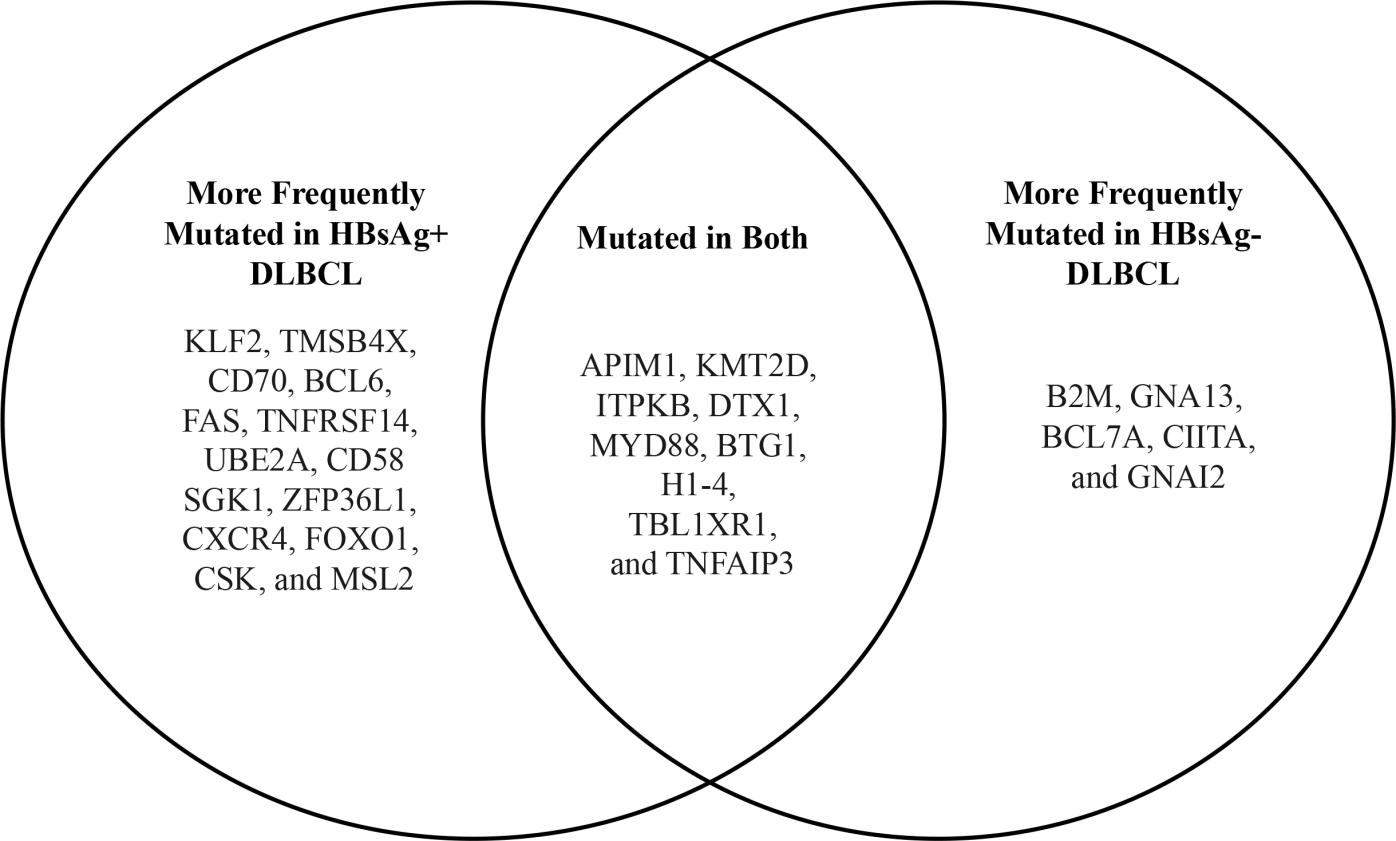
Comparison of mutated genes from HBsAg+ and HBsAg- DLBCL (data from Ren W. et al. (39)).

The mutational landscape of HBV-associated DLBCL in a cohort of 275 Chinese patients was assessed in a landmark 2018 study by Ren and colleagues (39). This study showed that DLBCL patients with concomitant HBV infection were characterized by a younger age (median age, 42 vs. 60 years; p <.0001), more advanced disease stage at diagnosis, and shorter overall survival. GC-type and ABC-type DLBCL were equally frequent. An enhanced rate of mutagenesis and an increased total mutation load were observed in HBsAg+ DLBCL genomes (median, 15,036 vs. 9,902 mutations). In addition, more non-silent mutations were observed in HbsAg+ DLBCLs (median, 99 vs 66). The genome-wide mutational signatures of 60 DLBCL cases were characterized based on the 96 possible mutation types. Seven mutational signatures were extracted from the cohort and three were significantly enriched in HBsAg+ tumors. One of the signatures was linked to APOBEC enzymes, a family of proteins with anti-viral functions (71), suggesting that HBV-associated DLBCLs are associated with distinct mutational signatures. Additionally, *TMSB4X, FAS, UBE2A, DDX3X, CXCR4, KLF2*, and *SGK1*, were significantly more mutated in the HBsAg+ group. Some of these genes are potential targets for activation-induced cytidine deaminase (AID), the driver of somatic hypermutation (SHM) in the immunoglobulin (Ig) genes during B-cell maturation and selection in the germinal center. The study also showed that the frequency of chromosomal translocations involving *BCL6* was significantly increased in HBsAg+ DLBCL genomes (57% vs 28%; p = .0472), suggesting that *BCL6* dysregulation plays a role in HBV-associated DLBCL. Finally, antigen processing and p53 signaling pathway-associated genes were significantly upregulated in HBsAg+ DLBCLs (**Figure 3**). These mutations or deletions carry a particularly poor prognosis for DLBCL. Interestingly, most of the genes that were mutated in HBV-associated DLBCLs were not mutated in HBV-associated HCC or HBV-positive lung adenocarcinoma, suggesting that genetic alterations found in HBV-associated DLBCL may be B-cell specific. The finding of APOBEC signatures and the lack of sequence homology of the CD3 region on characterization of the V(D)J region of immunoglobulin heavy chain (IgH) suggest that the lymphoma was due to direct carcinogenic effects of the virus rather than due to chronic antigen stimulation. Based on these observations, the authors proposed that HBsAg+ DLBCL should be considered and classified as a distinct subtype of DLBCL (**Table 4**).

**Table 4:** Summary of major genomic findings in HBV-positive DLBCL.

Study (Year)	Location	Sample	Key Findings
Huang X, Young KH, Guo W, et al. (2020) (16)	China	96 HBsAg+ and 10 HBsAg- DLBCL cases	The HBV antigen HBx, a protein essential for viral replication, was present in the lymphoma cells in 48.9% of HBsAg+ DLBCL patients; additionally, the HBV antigen Pre-S2, a component of HBsAg, was detected in the lymphoma cells of 57.2% HBsAg+ DLBCL patients. Notably, the authors also showed that the presence of HBx antigen in DLBCL cells was associated with high MYC expression (p = 0.0302). The frequency of MYC gene rearrangement was significantly higher in HBV+ DLBCL cases than in the HBV- group.
Ren W, Ye X, Su H, et al. (2018) (39)	China	275 DLBCL cases	An enhanced rate of mutagenesis and an increased total mutation load were observed in HBsAg+ DLBCL genomes. More non-silent mutations were observed in HbsAg+ DLBCLs (p = 0.048). *TMSB4X, FAS, UBE2A, DDX3X, CXCR4, KLF2*, and *SGK1*, were significantly more mutated in the HBsAg+ group (p < 0.05). The frequency of chromosomal translocations involving *BCL6* was significantly increased in HBsAg+ DLBCL genomes (57% vs 28%; p = .0472) Antigen processing and p53 signaling pathway-associated genes were significantly upregulated in HBsAg+ DLBCLs (p = 0.015 and 0.036, respectively), as were BCL6-trageted, ZFP36L1-bound, and FOXO1-bound genes (p = 0.008, 0.000, 0.002, respectively).

## 4 Open questions and future directions

Most of the sero-epidemiological studies that have assessed the link between B-cell NHL and HBV, and in particular the impact of chronic HBV infection on clinical outcomes, have been performed in East Asian countries, where HBV is endemic. Several environmental and host- or tumor-specific factors, however, may confound the strength of the association between HBV infection and clinical outcome in B-cell NHL, including DLBCL. In addition, while two positive meta-analyses have been published, the relatively limited sample size of each individual study (the largest study by Ren et al. had 275 patients) and the bias inevitably present in retrospective datasets make any final conclusion about the impact of HBV on survival and response to treatment in DLBCL premature. It is therefore of significant interest to determine if such findings can be confirmed in non-endemic countries, in populations of different racial and ethnic backgrounds, and ideally in larger studies. In terms of ascertaining relative risk, considering that the prevalence of HBV infection is not as high in the U.S., it will be more challenging to validate the higher prevalence of B-NHL among patients with HBV infection, compared to endemic countries. However, focusing on subsets of U.S. patients with a higher prevalence of HBV infection but not from endemic areas may mitigate this challenge. A large enough multi-center retrospective study in North America or Europe in an unselected DLBCL population could provide a dataset addressing whether the higher prevalence and inferior survival outcomes of patients with concomitant HBV and DLBCL observed in Asia can be confirmed. If a study in non-endemic areas were to strongly suggest or confirm the inferior clinical outcome of DLBCL patients with concomitant HBV, it would also be important to determine if these disparities affect specific subsets of DLBCL patients, based on patient characteristics (ethnicity, race, age, comorbidities) or subtype of DLBCL (cell-of-origin, double hit, or double expressor status) defined by the standard of care methods (IHC, FISH). These questions can be addressed by a retrospective study, since all patients diagnosed with B-NHL in the U.S., including those with DLBCL, are screened for prior HBV infection prior to initiating therapy, with serologies for HBsAg, HBsAb, and HBcAB.

Of even greater interest is whether the mutational spectrum and the prevalence and specificity of the genomic signatures described by Ren and colleagues in HBV-associated DLBCL can be confirmed, although such studies will require more resources and coordination among centers. Given the increase in mutagenesis observed in HBsAg+ DLBCL (39), it would be important to determine if there is an association between HBV and one or more of the genetic subtypes of DLBCL defined by Schmitz et al. (34) and Chapuy et al. (32) The off-target AID-associated mutagenesis observed in lymphoma cases with HBV integration and the BCL6 chromosomal translocations in HBV associated DLBCL (39) could potentially lead to a higher prevalence of DH/TH lymphomas among HBV associated DLBCL. By specifying the DLBCL subtypes most impacted by HBV, it might also be possible to propose a mechanism by which HBV infection contributes to the development of DLBCL, and more broadly, B-cell NHL. This could also elucidate new prognostic factors and aid in earlier detection, treatment, or prevention of NHL in chronic HBV patients. Finally, it remains unclear, even from the retrospective studies conducted in Asia, whether *past* HBV exposure (HBsAg-, HBcAb+) confers an increased risk of B-NHL and worse clinical outcomes for B-NHL, or whether the presence of chronic HBV infection (HBsAg+, HBcAb+) is what ultimately confers these risks (discussed below).

Because HBV virus antigens can be detected in DLBCL cells via IHC in HBsAg+ patients using formalin-fixed paraffin-embedded (FFPE) tissue (16), the clinical relevance of such findings can be assessed in retrospective studies, to determine whether the presence of HBV antigens in the lymphoma cells of HBV associated DLBCL leads to distinct clinical characteristics or outcomes. For example: assuming that a linkage between chronic (or past) HBV infection is confirmed, is the presence of HBV antigens in the lymphoma cells necessary to confer worse clinical outcomes? This will be essential in the design of prognostic tools based on HBV status and for developing personalized therapies for these patients.

In need of further investigation is also the issue of occult HBV infection (OBI), defined as a condition where replication-competent HBV DNA is present in the liver or other tissues, with or without detectable HBV DNA in blood or plasma, in *HBsAg-negative* patients. HBV genome sequences were recently detected by NGS in plasma, normal B-cells, and tumor tissues from 40 HBsAg-negative DLBCL patients, 27 of which (68%) had OBI (72). Sequencing of these gene segments revealed a high frequency of viral DNA variants, including a T1762A/A1764G missense mutation in the basal core promoter and an HBsAg missense mutation that could account for HBsAg negativity and therefore OBI status. The accumulation of viral variants could provide HBV with a survival advantage and drive lymphomagenesis in DLBCL in the absence of clinically overt chronic HBV infection. Additional support for the clinical impact of OBI in patients with lymphoma comes from studies showing that HBV reactivation in HBsAg-negative lymphoma patients receiving chemo-immunotherapy can occur (73–75), suggesting the need for antiviral prophylaxis in this group, and from a case-control study from Japan showing that patients with OBI had a higher prevalence of DLBCL than other groups (76).

Lastly, the association of HBV with indolent B-cell NHL might also be of interest, considering that HBV-associated follicular lymphoma (FL) was recently reported to also have distinct clinical and genetic features and worse outcomes (77–79).

## 5 Conclusions

The available evidence shows that chronic HBV infection is associated with a higher risk of developing B-cell NHL, particularly DLBCL, with odds ratios consistently ranging between 2.0 and 4.0. The relative risk of lymphoma associated with HBV infection is significantly lower compared to that of HCC, but, considering the global prevalence of HBV, it amounts to a very high burden of aggregate lymphoma risk. There is also evidence that DLBCL patients with chronic HBV infection have more aggressive disease, greater frequency of high-risk IPI, and inferior outcomes with R-CHOP, compared to patients without chronic HBV infection. Differential risk, clinical presentation, and outcome studies come almost exclusively from endemic areas, such as China, Taiwan, and Japan, and need to be confirmed in non-endemic settings. HBV-positive DLBCL showed a distinct gene expression profile, spectrum of somatic mutations, and genetic signatures enriched in pathways associated with high mutagenesis, suggesting that HBV-associated DLBCL are a distinct subtype. Finally, the risk of developing B-NHL in patients with OBI needs to be better studied considering the magnitude of the population at risk.

## Author contributions

MR: Conceptualization, Investigation, Methodology, Project administration, Resources, Writing – original draft, Writing – review & editing. MP: Conceptualization, Investigation, Methodology, Project administration, Resources, Writing – original draft, Writing – review & editing. CT: Conceptualization, Investigation, Methodology, Project administration, Resources, Writing – original draft, Writing – review & editing. PS: Conceptualization, Investigation, Methodology, Project administration, Resources, Writing – original draft, Writing – review & editing. AK: Conceptualization, Investigation, Methodology, Project administration, Resources, Writing – original draft, Writing – review & editing. PP: Conceptualization, Investigation, Methodology, Project administration, Resources, Supervision, Writing – original draft, Writing – review & editing.
